# Clinical Nurses Research Priorities in Hospital Settings: A Delphi Survey

**DOI:** 10.1177/01939459211017919

**Published:** 2021-05-27

**Authors:** Mariann Fossum, Marlene Z. Cohen, Vivi Haavik Tønnessen, Mette Dobler Hamre, Agno Lisbeth Vabo Ødegaard, Ingjerd Lind, Kari Olsen Håheim, Anne Opsal

**Affiliations:** 1 Centre for Caring Research—Southern Norway, University of Agder, Grimstad, Norway; 2 VA Nebraska-Western Iowa Health Care System, Omaha, NE, USA; 3 University of Nebraska Medical Center, Omaha, NE, USA; 4 Sorlandet Hospital, Kristiansand, Norway

**Keywords:** acute-care hospitals, evidence-based practices, nurses, priority research themes

## Abstract

This study aimed to identify the research priorities of clinical nurses to develop a research program at a health care services system that includes three hospitals. A Delphi survey was emailed to all clinical nurses in two rounds. The Delphi method was used to collect data from the nurses in regards to their priority research themes, and the data were analyzed using descriptive and comparative statistics. A total of 933 clinical nurses returned the first round of the Delphi survey and 543 nurses answered the second round. Clinical nurses identified 89 potential research themes. Patient safety and ethical challenges were the two highest ranked research priorities. The 40 highest ranked priority research themes were closely associated with issues concerning patient care and ethics. However, the nurses also gave high ratings to issues relating to the work environment, questions about technology implementation, and patient involvement in clinical care decisions.

Identifying research priorities centered on patient care may contribute to effective and efficient health care services (Al-Yateem et al., 2018; Schoenly, 2015; Struwe et al., 2018). Bedside nurses are well-positioned to identify the most important problems in patient care and to ask clinically relevant research questions (Wielenga et al., 2015). Thus, the clinical nurse plays a key role in the development of a research culture (Berthelsen & Hølge-Hazelton, 2017; Rytterström et al., 2009), and involving them in developing research priorities may increase their engagement in research and enhance quality development in nursing practice.

The development of a research culture in the clinical setting of acute-care hospitals is required to promote an evidence-based, clinical nursing practice and improve patient outcomes (Berthelsen & Hølge-Hazelton, 2017) and is characterized by having clinicians interested and motivated in research. However, clinical nurses and heads of hospital wards often lack research competencies (Bäck-Pettersson et al., 2008). A research culture is essential for the active involvement of nurses in the research process and clinical-practice research in the wards. Therefore, knowledge concerning the research priorities of clinical nurses may help to better understand how to develop a research culture in these clinical settings (Berthelsen & Hølge-Hazelton, 2017).

The Delphi survey method is particularly useful to reach consensus across stakeholders and the Delphi rounds continue until consensus is reached (Staykova, 2019). The Delphi survey method has been used in several studies with a focus on specialist settings. For instance, they have been used to determine the research priorities of nurses in different health care settings in countries such as Sweden (Bäck-Pettersson et al., 2008), Australia (Wilson et al., 2010), Canada (Lambert et al., 2019), Uganda (Spies et al., 2015), Hong Kong (Wong et al., 2019), the United States (Cohen et al., 2004; Jordan et al., 2016), the United Kingdom (Shepherd et al., 2017), Spain (Moreno-Casbas et al., 2001; Paz-Pascual et al., 2019), Ireland (Kelly, 2014), Iran (Hosseinzadeh et al., 2019; Oskouie et al., 2018), and the United Arab Emirates (Al-Yateem et al., 2019). A literature search identified over 50 Delphi studies that have been used to investigate the research priorities of nurses published between 1996 (Daly et al., 1996) and 2020 (Biccard & APORG, 2020). However, currently no Delphi study has been conducted in Norway. Health care priorities are highly context dependent; therefore, we determined there was a need for a Norwegian Delphi survey. We anticipated that data from a Delphi survey would provide valuable knowledge regarding the research priorities of clinical nurses in the acute-care hospital setting in Norway. These data can also be used for internal development of research and for further collaboration among local hospitals and the local university.

Previous Delphi surveys have focused on different areas of nursing practice such as oncology (Cox et al., 2017), neonatal intensive care (Wielenga et al., 2015), palliative care (Wong et al., 2019), nursing management (Sun & Prufeta, 2019), pediatrics (Williams et al., 2017), anesthesia nursing (Jordan et al., 2016), primary care (Evans et al., 2004), veteran nursing (Struwe et al., 2018), and acute-care hospital nursing (Al-Yateem et al., 2019). In these studies, nurses who were experts in their particular field were asked to identify research priorities within their area of practice. Engaging clinical nurses in research is vital to improve the quality of nursing research and increase their competency toward the application of research in clinical practice.

## Purpose

The purpose of this study was to identify the research priorities of clinical nurses at three hospitals in Southern Norway to develop a research program at a health care services system.

## Methods

In this descriptive study we used a Delphi technique in two rounds for clinical nurses at three hospitals to identify potential research priorities. The Delphi method is commonly used to collect expert opinions about real-world problems, and the method ensures that participants provide unbiased answers (McPherson et al., 2018; Staykova, 2019). In Delphi studies, the number of participants, how heterogeneous they are, and the candidate priority items generated can vary considerably; therefore, the technique can entail several data-collection stages to reach consensus across stakeholders and the Delphi rounds continue to consensus is reached. The number of participants and data-collection rounds is decided based on the purpose of the study (McPherson et al., 2018; Staykova, 2019). For this study, the population included all clinical nurses working in patient care at three hospitals. No additional inclusion criteria were used. As we aimed to explore areas of potential research priorities, two rounds were considered sufficient due to that consensus was researched in the second round.

### Study Design and Setting

Three local hospitals in two counties in Southern Norway were included in the study. All clinical nurses in all of the wards were asked about their research priorities in the first round of the study. The different wards involved were maternity and labor, general medicine, general surgery, pediatrics, emergency room, operating room, outpatient clinic, mental health, intensive care (ICU), and long-term care. The three hospitals were community based with 503 beds in the general wards and 57 ICU and recovery beds. The mental health clinic had 263 beds.

### Survey Procedure and Questionnaire

A major challenge was obtaining participation from 2,282 clinical nurses who worked in a variety of settings. A collaborative advisory group formed by a group of managers at the local hospitals and three researchers from two different universities were involved in planning and conducting the study. The advisory team discussed how to best reach the nurses. Two questionnaires were used to collect data in two consecutive rounds. While paper questionnaires have some benefits, the advisory team decided to use e-mail to send out the two Delphi questionnaires. The advisory team appealed to the hospital administrators to obtain the e-mail addresses for all of the nurses working in direct patient care. The hospitals had a strict protocol for sending e-mail questionnaires to employees, but the topic was considered important for the hospital and, therefore, the administrators agreed to e-mail the Delphi survey twice (the second e-mail after two weeks) and, in addition, emailed two reminders (after one month and six weeks).

The questionnaire used in round I came from a similar study conducted in the United States (Cohen et al., 2004), which was translated, reviewed and adapted to the Norwegian setting. It contained questions regarding demographic characteristics and one open-ended question: “What do you see as problems/issues in your ward that need to be studied?” Participants could add as many problems/issues as they wanted in free text. The questionnaire was pilot tested on four nurses and only minor text was changed. The electronic survey tool SurveyXact (Ramboll, Aarhus, Denmark) was used for the data collection. The questionnaire used in round II was developed specifically for the study based on the items the participants identified in Round I, as was done in prior Delphi studies (e.g., Cohen et al., 2004).

### Round I

The round I questionnaire was distributed to all 2,282 nurses via e-mail in April 2017. It was sent to clinical nurses at 60 wards at three different hospitals. Members of the advisory group divided the various areas of the hospital wards and took responsibility for informing the nurses in each area about the Delphi study. A number of strategies was used to inform nurses about the survey and encourage them to participate. This included attending staff and council meetings and having individual discussions with hospital staff. Information about the study was also published on the hospital’s internet site and repeated several times during the study period. The survey was voluntary and anonymous, however participating nurses were given the option to participate in a drawing for 10 gift certificates worth 300 Norwegian kroner (approximately $30 USD).

Demographic information and the identified patient care problems or issues were entered into a database by the researchers at the university. Descriptive statistics were used to summarize the demographic data. Responses to the open-ended question were content analyzed line-by-line and assigned a label to each priority. All responses were included in the analysis. Two researchers at the university who have extensive experience with qualitative analysis sorted the data for duplicate ideas and variations in phrasing. The advisory research team then analyzed the labels and each priority. A total of 89 research priorities were identified and grouped.

### Round II

In round II, nurses rated the priority of the 89 potential research items generated in round I. The questionnaire was only emailed to general medical and surgical wards, specialty wards, and outpatient clinics, and not to clinical nurses at the mental health wards. Thus, round II was emailed out to a slightly smaller group of nurses. The main reason for this change was that the research team had expertise only in the general medical and surgical wards, specialty wards, and outpatient clinics so the data from the mental health wards (Round I) were analyzed by a group of researchers from the mental health wards and not included in this analysis (Round II). Each item was scored on a scale of 1 (not-at-all important) to 5 (extremely important). The new questionnaire was distributed to 1,702 nurses by e-mail in October 2017. All surveys were returned to the two researchers at the university. As in round I, the round II Delphi survey was voluntary and anonymous, but the nurses were given the option to participate in a prize draw for 10 gift certificates of 300 Norwegian kroner.

### Data Analysis

The text from the potential research priorities identified in round I was clustered into thematic domains according to content using the framework suggested by Pope et al. (2000) using the following five stages; familiarization, identifying a thematic framework, indexing, charting, and mapping interpretation. In the first stage, two of the authors (MF, AO) read the entire text. In the next stage, single passages of text that contained several themes were divided into categories, with some passages including several categories. All of the data relevant to each category was examined, and condensed research items were rearranged and mapped to relevant themes. A focus was placed on keeping the participants’ original meaning.

We used Nvivo 12 software (QSR International, 2021) to facilitate the search for content. The research items identified in round I were grouped into the following 10 areas for the questionnaires in round II: management, organization, and patient safety (MOP), technology and digital competence (TD), communication (C), documentation (D), cultural sensitivity (CS), ethics (E), patient care (PC), quality improvement, competence raising and working environment (QCW), mental health and substance abuse (MS), and patient involvement (PI). Data from round II were assessed using principal component analysis, which aims to account for the variance in a measure to reduce the data into fewer, more manageable variables. The descriptive data were analyzed using SurveyXact (Rambøll, 2021). As the survey was anonymous, the institutional review board did not permit the team to link the two questionnaires. However, we believe that those who did not return the first survey were less likely to return the second one.

### Ethical Considerations

The study was approved by the Norwegian Center for Research Data, project number 52,110, and approved by the local hospitals and the university research ethics committee. The nurses did not sign an informed consent form due to the low-risk nature of study and to ensure anonymity. Data collection instruments for both rounds of the Delphi study included a statement describing the purpose of the research and that completing the questionnaire implied consent.

## Results

### The Study Groups

In round I, 2,282 surveys were emailed and 933 (41%) were returned. In round II, 1,694 surveys were emailed and 543 (32%) nurses responded. The average age for the nurses was 45 years (*SD*: 11.5) (range 20–66) in round I and 44.4 years (*SD*: 11.3) (range 22–65) in round II. The majority of the respondents worked in clinical practice and slightly more than half worked full-time. A total of 571 (61%) respondents in round I and 320 (59%) respondents in round II stated that they were not involved in research. Respondents who were involved in research collected consent forms, explained research projects to patients, developed research protocols, collected data for research projects, participated as informants or interviewers, and participated in data analysis. Table 1 displays the background characteristics of the respondents from the two rounds.

**Table 1.** Demographic Characteristics of Participants from the Delphi Study: Round I (*N* = 933) and II (*N* = 543).

Variable	Round I, *n* (%)	Round II, *n* (%)
Gender (*N* = 926/*N* = 539)		
Male	123 (13%)	40 (7%)
Female	810 (87%)	503 (93%)
Education (*N* = 914/*N* = 530)		
Bachelor’s degree	436 (47%)	280 (51%)
Master’s degree	95 (10%)	67 (12%)
Postgraduate education	377 (40%)	183 (34%)
PhD	6 (1%)	0 (0%)
Employment (*N* = 899/*N* = 522)		
Full time	541 (58%)	280 (52%)
Part time	302 (32%)	219 (40%)
Job role (several answers were possible)		
Clinical	750	451
Managerial/administrative	128	57
Quality	77	54
Other	88	34
Involvement in research		
(Several answers were possible)		
Collecting consent forms	125	88
Explain research projects to patients	100	65
Developing research protocols	10	4
Collecting data for research projects	116	60
Participating as an informant or interviewer	76	43
Participating in data analysis	24	17
Not involved in research	571	320
Other	97	48

### The Delphi Surveys

Round I of the Delphi survey resulted in 1,944 answers (which included many duplicate items), which were summarized in 89 potential research items. Table 2 presents the 10 types of research areas and examples of research areas. Round II identified the following top 10 research priorities: (1) patient safety, (2) ethical challenges in end-of-life treatment, (3) sepsis, (4) resuscitation, (5) staffing and workload, (6) pain management, (7) debriefing of staff working with the acute and critically ill, (8) work environment, (9) early warning scores, and (10) consequences of rotating work shifts. Table 3 presents the rank order of the top 40 research priorities by their mean ± standard deviation (*SD*) scores, which ranged from 4.5 to 4.0 (*SD*: 0.98–0.80). A total of 426 respondents had complete data for all 89 items.

**Table 2.** Type of Research Area and Examples of Research Areas.

Type of Research Area	Example of Research Area
Management, organization, and patient safety (MOP)	Reporting incidents Interdisciplinary collaboration Ward culture
Technology and digital competence (TD)	Medical-technical equipment Simulation eHealth
Communication (C)	Debriefing of staff working with the acute and critically ill Follow-up conversations with patient and relatives The duty of confidentiality
Documentation (D)	Nursing documentation—use of treatment plans Nursing minimum data set Comprehensive geriatric assessment
Cultural sensitivity (CS)	Cultural sensitivity and knowledge of other cultures Foreign-language speaking patients and use of interpreting Cultural attitudes of nurses toward patients from diverse cultures
Ethics (E)	Ethical challenges in end-of-life treatment Resuscitation Challenges with aggressive patient behavior
Patient care (PC)	Early warning score Wound care Nutrition support in the hospital
Quality improvement, competence raising and working environment (QCW)	Staff and workload Evidence-based practice Nursing-sensitive quality indicators
Mental health and substance abuse (MS)	Use of coercion Anxiety and restlessness Drugs, addiction, and intoxication
Patient involvement (PI)	Patient experience and patient satisfaction The patient participates in the choice of treatment and care Patient education and learning

**Table 3.** Rank Order of the Top 40 Research Priorities (*N* = 538).

Area	Type of Area	Rank	*N*	Mean	Standard Deviation
Patient safety	MOP	1	474	4.5	0.80
Ethical challenges in end-of-life treatment	E	2	456	4.4	0.81
Sepsis	PC	2	441	4.4	0.81
Resuscitation	E	3	456	4.4	0.82
Staff and workload	QCW	4	428	4.4	0.83
Pain management	PC	5	440	4.3	0.83
Debriefing of staff working with the acute and critically ill	C	6	456	4.3	0.85
Work environment	QCW	7	428	4.3	0.86
Early warning score	PC	8	448	4.3	0.87
Consequences of roaming work shift duty	QCW	8	428	4.3	0.87
Handling confidentiality	C	9	456	4.3	0.94
Competence-enhancing interventions and professional development	QCW	10	428	4.2	0.86
Burnout and stress	QCW	11	428	4.2	0.87
Challenges with dementia, troubled and aggressive patients	E	12	456	4.2	0.88
Medical-technical equipment	TD	13	456	4.2	0.90
Quality of life	PC	14	440	4.2	0.92
Reporting incidents	MOP	15	474	4.1	0.88
Complications associated with treatment	PC	16	447	4.1	0.86
Quality assurance of procedures	QCW	17	428	4.1	0.91
Interdisciplinary collaboration	MOP	18	474	4.1	0.92
Fighting sadness and bad news	E	19	456	4.1	0.92
Prevention of infections	PC	20	441	4.1	0.93
Relief of symptoms	PC	20	441	4.1	0.93
Follow-up conversations with patient and relatives	C	21	456	4.1	0.94
Culture in the ward	MOP	21	474	4.1	0.94
Medication management	PC	22	440	4.1	0.95
Patient information and preparation for examinations and treatment	QCW	23	428	4.0	0.93
Knowledge-based practice	QCW	23	428	4.0	0.93
Guidance and follow-up of students	QCW	23	428	4.0	0.93
Patient experience and patient satisfaction	PI	23	426	4.0	0.93
Patient progress and waiting challenges	MOP	23	473	4.0	0.93
Wound care	PC	24	440	4.0	0.94
Collaboration across wards	MOP	24	474	4.0	0.94
Prevention and treatment of delirium (acute confusion)	PC	24	447	4.0	0.94
Simulation	TD	25	456	4.0	0.95
Cultural sensitivity and knowledge of other cultures	CS	25	456	4.0	0.95
Nursing documentation—use of treatment plans	D	26	455	4.0	0.96
Absenteeism	QCW	26	428	4.0	0.96
The patient participates in the choice of treatment and care	PI	27	426	4.0	0.97
Violence and threats to employees	QCW	28	428	4.0	0.98

*Note*. Management, organization, and patient safety (MOP); technology and digital competence (TD); communication (C); documentation (D); cultural sensitivity (CS); ethics (E); patient care (PC); quality improvement, competence raising and working environment (QCW); mental health and substance abuse (MS); patient involvement (PI).

The three highest ranked potential research priorities from the list of top 40 research priorities were quality improvement, competence raising, and working environment (QCW), management, organization, and patient safety (MOP), and ethics (E).

## Discussion

To our knowledge, this is the first Delphi study conducted in Norway that explores potential research priorities identified by clinical nurses in acute-care hospitals. The purpose was to use the results as a basis for developing research programs at a health care-service system, and to identify a consensus of research priorities among the participants. The highest ranked priority in our study was patient safety, followed by ethical challenges in end-of-life treatment and sepsis. Patient safety was also the highest ranked in a study by Al-Yateem et al. (2019). Moreover, patient safety was ranked within the top five in two other Delphi studies (Struwe et al., 2018; Wilson et al., 2010). While clinical nurses work closely with patients, nearly half of the research priorities ranked in the top 10 were system and organizational topics. This is consistent with findings from three other Delphi surveys (Shepherd et al., 2017; Struwe et al., 2018; Sun & Prufeta, 2019) and may be explained by work issues experienced by clinical nurses. Moreover, a healthy work environment could have a direct impact on patient safety and outcomes (Copanitsanou et al., 2017; Wei et al., 2018). Therefore, more studies that focus on improving these system areas are indicated. Currently, studies being conducted in this area are primarily descriptive, so high-quality intervention studies should be considered (Wei et al., 2018).

Half of the research priorities in the top 10 were, not unexpectedly, clinical topics. However, it is worth considering whether these priorities reflect research priorities or educational needs of the nurses. A substantial amount of research exists for some of the items that were identified. Nurses could benefit from education on evidence-based practice on these items. This finding could be because of the broad focus of this exploratory Delphi study. In addition, all clinical nurses were included as experts so perceptions of inexperienced clinical nurses could have had an impact on this too. However, two other Delphi surveys (Al-Yateem et al., 2019; Schoenly, 2015) have also discussed the issue regarding research priorities versus educational needs.

Nurses need to use the best available knowledge when making clinical decisions (Nibbelink & Brewer, 2018). Therefore, the study results were emailed to head administrators at the wards involved in the study. Clinical nurses who participated in this research could be better prepared for providing evidence-based care, thus, engaging in research is important to improve decision-making and outcomes for patients. This survey was an important first step in the process of changing nursing culture to increase research and evidence-based practice, and our results can be used to guide the development of research programs in acute-care hospitals.

Some limitations include that the study is conducted in one county in Norway, reducing the opportunities to generalize the findings. In addition, SurveyXact was used for data collection to allow easier access; however, some nurses did not check their e-mail regularly, which was a disadvantage of this approach and another limitation.

This survey was organized by the participating hospitals and included email reminders. However, a low response rate is common in Delphi surveys (Staykova, 2019), and this study had a response rate of 41% and 32% for rounds I and II, respectively. Results were discussed in the advisory group, and the anonymous data per ward were provided to the individual wards following the analysis to encourage and establish a nursing research culture that includes research and evidence-based practice in the hospitals.

Overall, the implementation of evidence-based practice in nursing practice remains low (Craig & Dowding, 2019), and respondents in this study reported that they were not involved in research. Having limited opportunities for nurses to be involved in research activities could explain the low response rate in this study. Drop-out between the rounds is a limitation of the Delphi method (Staykova, 2019). However, the overall number of participants was good given that the purpose of the study was exploratory. The low response rate can be considered fair and perhaps participants who responded believed they had something to contribute and were interested in the area of nursing research and evidence-based practice.

In conclusion, this Delphi study of nursing research priorities obtained from acute-care hospitals in Norway displays common research priorities when compared with those from surveys in other countries. Overall, nurses listed numerous research priorities, and the 40 highest ranked ones were closely associated with issues dealing with patient care and ethics. However, nurses also gave high rankings to the working environment, questions about technology implementation, and patient involvement. Sharing these results among key stakeholders in acute-care hospitals could contribute to building a research culture and to inspire nurses to participate in local projects to address some of these research priorities. However, building research capacity is also important for the further development of a research culture and also needs to be taken into consideration. We suggest that further research should identify potential research priorities from the perspectives of patients and next-of kin, in addition to policymakers and other members of interdisciplinary teams in different types of hospitals.
